# Multidisciplinary assessment of tako tsubo cardiomyopathy: a prospective case study

**DOI:** 10.1186/1471-2261-11-14

**Published:** 2011-04-09

**Authors:** Micael Waldenborg, Mona Soholat, Anders Kähäri, Kent Emilsson, Ole Fröbert

**Affiliations:** 1 Department of Cardiology, Örebro University Hospital, Örebro, Sweden; 2 Department of Clinical Physiology, Örebro University Hospital, Örebro, Sweden; 3 Department of Psychiatry, Örebro University Hospital, Örebro, Sweden; 4 Department of Radiology, Orebro University Hospital, Orebro, Sweden

## Abstract

**Background:**

The cause of tako tsubo cardiomyopathy remains unclear. We used a multidisciplinary approach to investigate if a common pathophysiological denominator could be outlined.

**Methods:**

Within 3 days following symptom presentation and again after 3 months we investigated all patients coming to our institution and diagnosed with tako-tsubo cardiomyopathy. Patients underwent extensive biochemical screening. Left ventricular function was evaluated by echocardiography and contrast-enhanced cardiac magnetic resonance imaging. Cardiac autonomic function was studied by heart rate variability and signal-averaged electrocardiogram and posttraumatic stress and depression were investigated by questionnaires (the Posttraumatic Stress Syndrome 10-Questions Inventory, PTSS-10 and the Montgomery-Åsberg depression rating scale, self rated version, MADRS-S).

**Results:**

During 2 years, 13 consecutive patients were included. Markers of myocardial damage and heart failure were slightly to moderately elevated and ejection fraction (echocardiography and MRi) was moderately reduced at hospitalization and improved to normal values in all patients. Signal averaged ECG demonstrated a statistically significant shorter duration of the filtered QRS complex in the acute phase as compared to follow-up. In heart rate variability analysis, SDNN and SDANN were shorter acutely compared to follow-up. Two patients fulfilled criteria for posttraumatic stress syndrome while 7 patients were in the borderline zone. There was a statistically significant inverse correlation between PTSS-10 score and QRS duration in the signal-averaged ECG (r = -0.66, P = 0.01).

**Conclusions:**

Patients with tako tsubo cardiomyopathy have altered cardiac autonomic function and a high incidence rate of borderline or definite posttraumatic stress syndrome acutely. This is in line with findings in patients with myocardial infarction and does not allow conclusions on cause and effect.

## Background

Tako-tsubo cardiomyopathy is a rapidly resolving condition of unknown etiology. The typical patient is female, postmenopausal and present with symptoms, electrocardiographic (ECG) and clinical findings suggestive of ST-elevation myocardial infarction. However, coronary arteriography is normal or findings are unrelated to the often severe transient heart failure involving hypokinesia of the left ventricular mid segments and apical ballooning [1].

Tako tsubo cardiomyopathy has been associated with acute emotional stress [2] but this is not obligate [3] and recently it was hypothesized that changes in autonomic control of the cardiovascular system contribute to tako tsubo [4]. Other prominent hypotheses on pathophysiology postulate increased release or sensitivity to catecholamines [5] or alteration of Ca^2+^-handling proteins [6].

The purpose of this study was to offer a comprehensive multidisciplinary approach to all patients presenting with tako tsubo cardiomyopathy in our institution during a 2-year time period in order to better understand and treat the disease. We hypothesized a relation between scores of emotional stress and depression on one side and cardiological, physiological and biochemical measures of disease severity, on the other. In order to address our hypothesis we used an array of conventional cardiac examinations, scores of posttraumatic stress and heart rate variability and signal-averaged ECGs to assess ventricular late potentials at baseline and after 3 months.

## Methods

All patients suspected of having ST-elevation myocardial infarction and referred to our institution for acute for coronary arteriography between April 2008 and March 2010 were screened for the study. Criteria for inclusion were in line with the recently published Mayo clinic criteria for tako tsubo cardiomyopathy [7]: hypokinesis, akinesis, or dyskinesis of the left ventricular mid segments with or without apical involvement; absence of obstructive coronary disease or angiographic evidence of acute plaque rupture; new ECG abnormalities (either ST-segment elevation and/or T-wave inversion) and no clinical suspicion of pheochromocytoma or myocarditis. Patient screening and -inclusion was done in the catheterization laboratory following coronary arteriography and left ventriculography. All patients fulfilling the above criteria and giving written informed consent were extensively investigated. We recorded ECGs including assessment of heart rate variability (HRV) and signal-averaged ECGs (SAECG) to measure ventricular late potentials and extensive biochemical profiling including catecholamines was carried out. Left ventricular function was evaluated by echocardiography and cardiac magnetic resonance imaging (MRi) was used for assessment of cardiac function and myocardial viability.

In the acute phase and at the 3-months follow-up HRV was recorded using a battery operated solid-state recorder (DXP1000, Braemar, Eagan, USA) for 24-hour continuous recording of ambulatory ECG data and analyzed using an Aspect Holter system (Danica Biomedical, General Electric, Fairfield, United States). The following time-domain variables were calculated: average R-R interval value calculated from accepted beats (mean RR), standard deviation of all the NN (normal-to-normal) R-R intervals (SDNN), and the baseline width of the minimum square difference triangular interpolation of the highest peak of the histogram of NN intervals (TINN). The long-term HRV was quantified by the standard deviation of all 5-min NN interval mean values (SDANN). NN50 count, that is the number of interval differences of successive normal-to-normal intervals greater than 50 ms as well as SDSD, that is the mean of standard deviations of normal RR intervals for all 5-minute segments, were also calculated. Finally, the beat-to-beat HRV was estimated by the square root of the mean squared differences of successive NN intervals (RMSSD), and the ratio of NN interval differences of successive NN intervals greater than 50 ms to the total number of NN intervals (pNN50) [8].

Power spectral analysis was performed on the RR-interval data by means of fast Fourier transformation. The following components were measured: very low frequency (VLF), low frequency (LF), high frequency (HF) and the total power.

The SAECGs were obtained using a Megacart ECG recorder (Siemens-Elema AB, Solna, Sweden). In total, 300 beats were analysed using a noise reduction of 0.3 μV and a correlation of 0.98. The bipolar X, Y and Z lead system was used. The duration of the filtered QRS-complex (fQRS), the root mean square voltage, that is the RMS-amplitude during the last 40 ms of the filtered QRS-complex (RMS40) and the low amplitude signal, that is the time during which the amplitude of the filtered QRS-complex last part remains below 40 μV (LAS40) [8].

A Vivid 7 ultrasound machine (GE Vingmed Ultrasound A/S, Horten, Norway) equipped with a multi-frequency phased array transducer (M3S, 1.5-4.0 MHz) was used for the echocardiographic examinations and measurements were made after the examination using stored images on an EchoPac workstation (GE Vingmed). The subjects were examined in the left lateral recumbent position. Left ventricular ejection fraction (LVEF) was measured using the biplane Simpson's method

MRi was done with a Philips Achieva 1.5-T scanner. Standard bTFE (balanced Turbo Field Echo) cine-images were acquired as single 8 mm slice in long-axis, 3-chamber and 4-chamber view. Full ventricular coverage was achieved with 15-20 short-axis slices of 5 mm thickness. A delayed enhancement protocol was used 15 minutes after intravenous injection of Gadiodiamid (0.15 mmol/kg) with breath-hold inversion-recovery TFE using 10 slices of 10 mm thickness in long-axis, 3-chamber and 4-chamber view. About 15 slices were acquired in short-axis to get full ventricular coverage.

Patients answered questionnaires on posttraumatic stress (the Posttraumatic Stress Syndrome 10-Questions Inventory, PTSS-10) and depression (Montgomery-Åsberg depression rating scale, self-rated version, MADRS-S). The PTSS-10 is a self-administered questionnaire that measures the current presence of specific posttraumatic stress symptoms. Patients are asked to rate the presence of each symptom during the past 7 days and its severity on a scale from 1 (never) to 7 (always). A total score > 35 is associated with a high probability that the diagnostic criteria for post-traumatic stress disorder are fulfilled and a score between 27 and 35 is borderline [9]. The MADRS-S is an observer rating scale that records 10 depression items between 0 and 6, according to their intensity. A score > 34 is associated with major depression on the MADRS-S while 20-33 is considered borderline [10]. Both rating scales are available in Swedish.

All blood sampling was done in the morning after admission to the hospital, echocardiography within 24 hours and all other testing within 72 hours after admission. Most tests were repeated at a 3 months clinical control. The study protocol was approved by the Regional Ethical Committee in Uppsala, Sweden.

Statistical analysis was performed using SigmaStat 3.5 software (Systat, San Jose, Ca). Most variables were not normally distributed and we therefore used the Wilcoxon signed rank test for pairwise comparisons and Spearman's rank correlation coefficient for non-parametric assessment of statistical dependence between two variables. Differences were considered statistically significant when P < 0.05.

## Results

In the 2-year study period, between April 2008 and March 2010, 15 patients, all women, fulfilled criteria for inclusion. Two patients declined participation and 13 patients were included in the study (Table 1). All patients were treated with beta adrenergic receptor antagonists and angiotensin-converting-enzyme inhibitors between the acute phase and the 3-months follow-up.

**Table 1:** Baseline data of the patients (n = 13)

*Variable*	Median or number
Age, years	70 (69-74)
Women, n (%)	13 (100)
Height, cm	158 (152-163)
Weight, kg	65 (55-80)
Diabetes mellitus, n (%)	0 (0)
Current smoker, n (%)	1 (8)
Family history of IHD, n (%)	3 (23)
Definite trauma within 2 weeks, n (%)	6 (46)

Values are median (interquartile range) or number (percentage).

At arrival in the hospital, 9 patients had ST-segment elevation in two or more leads, 3 patients had poor R-wave progression and concomitant T-wave changes in the precordial leads and one patient had precordial ST-segment depressions. Markers of myocardial damage and heart failure were slightly to moderately elevated in all patients (Table 2). Catecholamines and thyroid parameters were normal and did not change from initial hospitalization to the 3-months control visit (although noradrenalin was borderline significant). Average cholesterol values were slightly above reference level and did also not change during follow-up (Table 2).

**Table 2:** Biochemical parameters and findings

*Variable*	Initial findings	48 hours	3 months	P
Troponin I (mg/L)	2.70 (0.45-2.90)	0.42 (0.17-0.76)	Not measured	< 0.001
Creatine kinase μkat/L	2.55 (1.58-3.15)	1.50 (1.00-1.90)	Not measured	0.03
Creatin kinase MB (μg/L)	11.60 (5.35-22.33)	4.40 (2.80-5.10)	Not measured	0.002
NT pro-brain natriuretic peptide (ng/L)	389 (228-447)	313 (275-702)	Not measured	0.32
High-sensitivity C-reactive protein (mg/L)	4.10 (2.90-6.70)	10.50 (7.10-19.60)	Not measured	0.02
Thyroid-stimulating hormone (mIU/L)	1.20 (0.68-1.70)	Not measured	1.10 (0.90-1.50)	0.97
Thyroxine (pmol/L)	14.30 (13.60-15.50)	Not measured	14.00 (13.30-15.00)	0.57
Cholesterol (mmol/L)	6.10 (5.30-6.60)	Not measured	5.40 (4.40-6.00)	0.57
Low-density lipoprotein cholesterol (mmol/L)	3.60 (2.70-4.40)	Not measured	3.00 (2.10-3.50)	0.52
High-density lipoprotein cholesterol (mmol/L)	1.50 (1.20-1.50)	Not measured	1.40 (1.30-1.60)	0.18
Triglyceride (mmol/L)	1.60 (1.20-2.20)	Not measured	1.50 (1.20-2.40)	0.41
Plasma adrenalin (nmol/l) *	0.30 (0.30-0.40)	Not measured	0.30 (0.30-0.30)	1.00
Plasma noradrenalin (nmol/l) *	3.70 (2.60-5.50)	Not measured	3.30 (2.50-3.40)	0.06

Values are median (interquartile range) or number (percentage).

*Complete data from 7 patients only

Of the 13 study patients, in 11 the left ventriculogram demonstrated apical ballooning and hypercontractile basal segments. In one patient a left ventriculogram was not performed but acute echocardiography demonstrated a similar pattern. In one patient only did the ventriculogram demonstrate an inverse pattern - mid-ventricular ballooning and apical akinesia. Ejection fraction was moderately reduced at hospitalization as demonstrated by both echocardiography and MRi and improved to normal values for all patients (Table 3). MRi could not be done in four patients (one had pacemaker, one elevated serum creatinine and two patients suffered from claustrophobia) but in none of the 9 patients undergoing MRi did late gadolinium enhancement demonstrate signs of myocardial necrosis.

**Table 3:** Data of left ventricular function

*Variable*	Initial findings	3 months	P
Ejection fraction, echocardiography (Simpsons method), %	51 (46-55)	65 (59-66)	0.003
Ejection fraction, magnetic resonnance imaging, %, *	50 (54-62)	73 (66-74)	0.02
End-diastolic volume, magnetic resonnance imaging, ml, *	134 (120-143)	129 (117-140)	0.06
End-diastolic diameter, magnetic resonnance imaging, mm*	41 (46-51)	44 (44-46)	0.56

Values are median (interquartile range).

*Complete data from 9 patients only

Signal averaged ECG recordings demonstrated a statistically significant shorter duration of the filtered QRS complex in the acute phase as compared to 3-months follow-up (despite the fact that the median values were almost identical, Table 4). Also in the acute phase there was a trend towards reduced root mean square voltage of the signal in the last 40 ms of the QRS complex (RMS 40) and of the low amplitude signal under 40 μv (LAS 40), but these parameters did not reach statistical significance.

**Table 4:** Data of late potentials and heart rate variability

*Late potentials*	Initial findings	3 months	P
fQRS, ms	104 (96-108)	103 (99-113)	0.01
RMS 40, mV	31 (16-38)	30 (10-34)	0.13
LAS 40, ms	31 (27-40)	35 (30-46)	0.06

***Heart rate variability***	**Initial findings**	**3 months**	**P**

Total, ms²	1300 (764-2241)	1881 (1276-2065)	0.24
VLF, ms²	929 (563-1463)	948 (875-1352)	0.34
LF, ms²	207 (154-469)	315 (186-558)	0.17
HF, ms²	155 (60-216)	132 (109-519)	0.59
SDNN, ms	91 (75-126)	114 (105-146)	0.008
SDANN, ms	78 (63-111)	103 (89-138)	0.008
RMSSD, ms	28 (21-35)	24 (22-41)	0.59
SDNN index, ms	41 (31-51)	47 (40-49)	0.17
SDSD, ms	28 (21-35)	24 (22-41)	0.59
NN50 count	4157 (967-5085)	2913 (1448-12500)	0.41
pNN50, %	3.96 (2.38-6.39)	3.02 (1.66-14.47)	0.50
TINN, ms	390 (290-600)	480 (400-610)	0.06

Values are median (interquartile range) or number (percentage). Abbreviations are: fQRS = duration of filtered QRS complex. RMS 40 = Root mean square voltage of signal in last 40 ms of QRS. LAS 40 = Low amplitude signal under 40 μv. VLF = very low frequency, LF = low frequency, HF = high frequency. SDNN = standard deviation of the normal-to-normal interval. SDANN = standard deviation of sequential 5-min heart period mean values. RMSSD = mean squared differences of successive normal-to-normal intervals. SDNN index = mean of standard deviations of normal RR intervals for all 5-minute segments. SDSD = standard deviation of successive differences. NN50 count = the number of interval differences of successive normal-to-normal intervals greater than 50 ms. pNN50 = percentage of adjacent normal-to-normal interval differences > 50 ms. TINN = triangular interpolation of RR intervals.

In HRV analysis, two time domain parameters came out statistically significant: standard deviation of the normal-to-normal interval (SDNN) and the standard deviation of sequential 5-min heart period mean values (SDANN) which were both shorter acutely as compared to three months follow-up (table 4). No other HRV parameter differed between the two time points.

**Figure 1:**
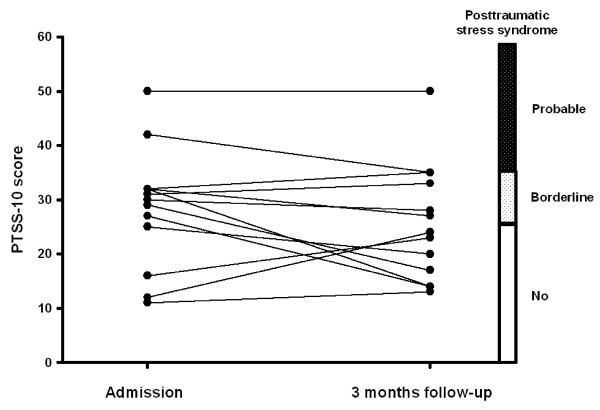
**Individual Posttraumatic Stress Syndrome 10-Questions Inventory (PTSS-10) scores at admission to the hospital after being diagnosed with tako tsubo cardiomyopathy and after 3 months follow-up**. Scoring criteria for posttraumatic stress syndrome are illustrated on the right-hand y-axis. There was not a statistically significant difference between PTSS-10 score at the two occasions (P = 0.30).

**Figure 2:**
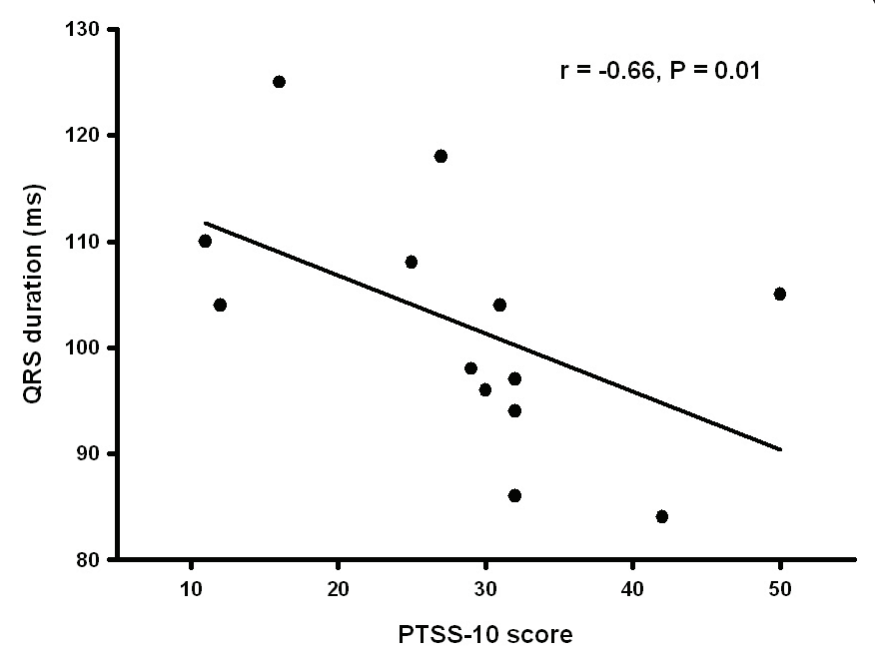
**Inverse correlation between Individual Posttraumatic Stress Syndrome 10-Questions Inventory (PTSS-10) scores at admission to the hospital after being diagnosed with tako tsubo cardiomyopathy and QRS duration in the signal-averaged ECG**.

Only one patient had a history of mental illness. This patient had been diagnosed with manio-depressive psychosis years before being admitted with tako tsubo. Her manio-depressive disease was considered stable at admittance and she scored 31 and 33 in the PTSS-10 scale (borderline) while the score was 13 on MADRS-S scale (no depression). At admission, 2 patients fulfilled criteria for posttraumatic stress syndrome while 7 patients were in the borderline zone. At 3 months follow-up one patient still fulfilled criteria for posttraumatic stress syndrome while 4 had borderline scores (Figure 1). We tested associations between PTSS-10 score and major variables of cardiac function (EF, NT pro-brain natriuretic peptide, TnI, adrenalin, noradrenalin, late potentials in the ECG and heart rate variability parameters). There was a statistically significant inverse correlation between PTSS-10 score and QRS duration in the signal-averaged ECG (r = -0.66, P = 0.01, Figure 2). Using the MADRS-S score one patient was in the borderline zone for depression acutely while all patients had scores within the normal range at follow-up. For the entire patient group there were no statistically significant differences in rating scale scores (PTSS-10 or MADRS-S) in the acute phase compared to 3-months follow-up (Table 5).

**Table 5:** Results of stress and depression rating scales

*Rating scale*	Initial findings	3 months follow-up	P
PTSS 10 questionnaire	30 (25-32)	24 (17-33)	0.3
MADRS questionnaire	8 (4-10)	10 (5.5-11)	0.49

Values are median (interquartile range). Abbreviations are: PTSS 10 = Posttraumatic Stress Syndrome 10-Questions Inventory. MADRS = Montgomery-Åsberg depression rating scale.

## Discussion

The main findings of this study on consecutive patients with tako tsubo cardiomyopathy were that in addition to clinical and laboratory indices of heart failure, cardiac autonomic function was altered and we found a relation between emotional stress and QRS duration.

Although the signal-averaged ECG values were within the limits of previously published normal values [11] we did find a significantly shorter duration of the filtered QRS in the acute phase compared to recovery. Filtered QRS duration is under influence of autonomic tone [12] but is it is not likely that the beta blocker treatment introduced in all patients influenced this parameter [13]. Filtered QRS duration has been associated with ventricular arrhythmias in non-ischemic heart failure [14], with ventricular asynchrony in chronic heart failure patients [15] and is reduced in healthy subjects to mental stress [16]. The finding of reduced filtered QRS duration acutely despite reduced LVEF and presumable susceptibility to ventricular arrhythmias in tako tsubo patients [17] in our view supports that emotional stress had more impact on this parameter than LV dysfunction.

HRV measured shortly after coming to the hospital with acute symptoms and again after recovery at 3 months demonstrated lower values of the time domain parameters SDNN and SDANN acutely. A German group recently made similar findings on HRV parameters [18] and the same group also published data that apical and midventricular variants of tako tsubo patients differ in some key HRV variables [19]. SDANN reflects both a sympathetic and parasympathetic heart rate modulation while a depressed SDANN is thought to indicate a relative sympathetic dominance [20]. In studies of heart failure patients SDNN and SDANN have been found to correlate inversely with plasma endothelin-1 [21] and noradrenaline [22] while acute pain had no effect on SDNN in a study of healthy male persons [23]. In a study of 138 patients in the early phase of acute myocardial infarction Doulalas et al. found similar SDNN values as we did in the present study (mean SDNN: 86 ± 35, SDANN: 74 ± 29) [24]. Emotional stress has been seen as a major contributor to causing tako tsubo cardiomyopathy and emotional stress also affects HRV. In one study a mental stress task significantly reduced SDNN index in patients after myocardial infarction [25]. In this study there was a statistically insignificant trend towards reduced SDNN index in the acute phase. Taken together, these different findings make it difficult to draw a firm conclusion on the acute SDNN reductions in our study; they might be a product of temporary heart failure or caused by emotional stress or even a blend of the two.

Posttraumatic stress is not uncommon in patients following myocardial infarction and was reported in 22% of patients in one study [26]. Thus, we cannot conclude that our findings of posttraumatic stress syndrome in two patients and borderline posttraumatic stress syndrome in another 7 support that psychological stress has a say in causing tako-tsubo in these patients. As with myocardial infarction patients, psychological stress in patients with tako tsubo cardiomyopathy can be secondary to chest pain and acute heart failure. It might have been more relevant if the patients had been examined for acute stress disorder. However, the information collected on the psychiatric surveys did not provide enough information to determine this.

Biochemical markers of myocardial damage were slightly to moderately elevated in the present study and this corresponds with previous findings in patients with tako tsubo cardiomyopathy [2,27]. In contrast with an earlier study [5] we did not find elevation in plasma catecholamines. However, we took only one blood sample and the routine use of intravenous diazepam before catheterization in our institution might have reduced catecholamine excretion [28]. Furthermore, ejection fractions as measured by echocardiography (recently published in detail elsewhere [29]) and MRi were reduced only moderately compared with findings by others [2,27]. We have no definite explanation to this difference; the timing of examinations in our study was identical to the previous ones and in line with these studies ejection fraction improved to normal.

Although the bulk of literature on tako tsubo cardiomyopathy is confined to case reports this study is limited by its size and by the fact that data come from a single center. Only a subset of patients was examined by MRi and had plasma catecholamine investigations but complete data on both parameters are unlikely to have changed our overall conclusion. Nevertheless, to our knowledge, this study is one of the most comprehensive and detailed investigations of patients with tako tsubo cardiomyopathy and the novel combined assessment of scores of emotional stress and HRV parameters seems clinically relevant and might inspire future studies of this disease.

## Conclusions

We conclude that patients with tako tsubo cardiomyopathy, in addition to clinical and laboratory indices of heart failure, have altered cardiac autonomic function and a high prevalence of borderline or definite posttraumatic stress syndrome. This is in line with findings in patients with myocardial infarction and does not allow conclusions on cause and effect in tako tsubo cardiomyopathy. In our opinion terms such as "stress-induced cardiomyopathy" or "broken heart syndrome" for this condition should be avoided.

## Competing interests

The authors declare that they have no competing interests.

## Authors' contributions

MW performed echocardiography, data management and analysis. MS participated in the design of the study and patient inclusion. AK designed the MRI investigations and did the MRI analyses. KE participated in study design, echocardiography and helped to draft the manuscript. OF designed the study, performed coronary angiographies, statistical analyses and drafted the manuscript. All authors read and approved the final manuscript.

## Pre-publication history

The pre-publication history for this paper can be accessed here:

http://www.biomedcentral.com/1471-2261/11/14/prepub
